# Mindfulness-based interventions in psychopedagogy for students with Attention Deficit Hyperactivity Disorder (ADHD): a scoping review

**DOI:** 10.3389/fpsyg.2026.1844428

**Published:** 2026-08-11

**Authors:** Jessica Vanessa Yépez-Verduga, Carolina Daysi Villacis-Macias

**Affiliations:** Universidad Estatal de MIlagro, Milagro, Ecuador

**Keywords:** academic performance, attention regulation, emotional regulation, executive function, mental health in education, special education

## Abstract

**Introduction:**

Mindfulness-based interventions have gained increasing relevance as complementary strategies for addressing attentional, behavioral, and emotional difficulties in individuals with Attention Deficit Hyperactivity Disorder (ADHD). This study aimed to systematically map and analyze the scientific evidence on mindfulness-based interventions in populations with ADHD, focusing on their effects on attention, academic performance, and emotional well-being, as well as on methodological limitations and research gaps.

**Methods:**

A scoping review was conducted following the PRISMA-ScR guidelines and the methodological recommendations of the Joanna Briggs Institute. Searches were performed in Scopus and Web of Science for original and review articles published in English or Spanish. The Population–Concept–Context framework guided the eligibility criteria. After duplicate removal and a structured screening of titles, abstracts, and eligible reports, 64 studies were included. Data were synthesized using descriptive, narrative, bibliometric, and thematic mapping approaches.

**Results:**

The evidence indicates that mindfulness-based interventions may improve attentional regulation, executive functioning, academic engagement, emotional control, coping strategies, and psychological well-being among individuals with ADHD or related attentional difficulties. Neurocognitive studies also reported functional changes in brain regions associated with attention and emotional regulation. However, the findings were not consistent across all outcomes, particularly regarding core symptoms of inattention and hyperactivity. Important limitations included small sample sizes, heterogeneous intervention protocols, variable methodological quality, adherence difficulties, and limited longitudinal evidence.

**Discussion:**

Mindfulness-based interventions represent promising complementary strategies for educational, psychopedagogical, and clinical settings, particularly for supporting attention and emotional self-regulation. Nevertheless, their effectiveness depends on context-sensitive implementation, participant adherence, and appropriate adaptation to the characteristics of ADHD. Future studies should use standardized protocols, larger and more diverse samples, longitudinal designs, and stronger evidence from underrepresented regions, particularly Latin America.

## 1 Introduction

In recent decades, interest in mindfulness-based interventions has grown steadily in both educational and clinical settings, particularly among populations with attentional difficulties such as Attention Deficit Hyperactivity Disorder (ADHD). This approach has become a relevant alternative for addressing problems associated with inattention, impulsivity, and emotional self-regulation—dimensions that directly affect academic performance and psychosocial well-being (Murrell et al., 2015).

Empirical evidence suggests that structured meditative practices can improve the cognitive processes underlying attention. In particular, interventions based on Samatha meditation have shown significant increases in academic engagement and performance on specific tasks, demonstrating positive effects on attentional regulation and executive function (Singh et al., 2016, 2018). These findings are complemented by clinical studies that integrate mindfulness within broader therapeutic approaches, reporting reductions in psychological symptoms and improvements in coping skills in individuals with ADHD (Santonastaso et al., 2020).

However, the implementation of these interventions presents significant challenges. In educational contexts, especially among university students, limitations related to adherence to programs, variability in intervention designs, and the cognitive characteristics of ADHD have been identified, which condition the effectiveness of the results (Murrell et al., 2015). Added to this are contemporary factors such as overexposure to digital environments, which have been associated with declines in concentration and sustained attention, reinforcing the need for strategies that strengthen cognitive control (Özbay, 2026).

From a neuroscientific perspective, neuroimaging techniques have enabled the identification of functional changes in brain regions linked to attentional control and emotional regulation, providing evidence of the neurobiological mechanisms that underpin the benefits of mindfulness (Fassbender et al., 2017b). Nevertheless, recent reviews warn of persistent methodological limitations, including small sample sizes, heterogeneity in approaches, and lack of standardization in protocols, which hinder the generalization of findings (Alorani et al., 2026; Ross et al., 2026).

In Latin American contexts, the evidence is still limited, although some studies have documented positive effects on the improvement of attention and emotional regulation in educational settings, suggesting relevant potential for implementation in these scenarios (Pérez Carmona, 2017). However, the scarcity of systematic research prevents the establishment of solid conclusions regarding their effectiveness in these contexts.

Within this framework, there remains a need to integrate and critically analyze the available evidence to identify trends, research gaps, and opportunities for application.

Consequently, the objective of this study is to systematically analyze the scientific evidence on mindfulness-based interventions in populations with ADHD, with emphasis on their effects on attention, academic performance, and emotional well-being, as well as on methodological limitations and implications for their implementation in educational and clinical contexts.

## 2 Methodology

### 2.1 Study design

The present study was developed using a scoping review approach, following the methodological guidelines proposed by Arksey and O’Malley, later refined by Levac et al., and reported in accordance with the PRISMA-ScR guide. This design is appropriate for systematically mapping the existing scientific literature on mindfulness-based interventions in psycho-pedagogical contexts aimed at students with Attention Deficit Hyperactivity Disorder (ADHD), identifying research trends, characteristics of the interventions, contexts of application, reported outcomes, and gaps in the evidence. This formulation follows the methodological logic of the studies by Guillen-Godoy et al. (2025) and Caranqui-Encalada et al. (2026), which also structure the review as a systematic mapping of the field, with emphasis on thematic trends, methodological approaches, and research gaps.

### 2.2 PCC eligibility framework

To delimit the scope of the review, the PCC framework (Population, Concept, Context) was used, widely recommended in scoping reviews:

- Population (P): students, children, adolescents, or young people with a diagnosis of ADHD, associated symptomatology, or school/psycho-pedagogical identification of attentional and hyperactivity difficulties.
- Concept (C): mindfulness-based interventions, mindfulness, mindfulness meditation, mindfulness-based programs, mindfulness training, or related strategies aimed at attentional, emotional, or behavioral self-regulation.
- Context (C): psycho-pedagogical, educational, school, clinical-educational, or learning support contexts, including programs implemented in schools, classrooms, psycho-pedagogical offices, special education, or psychoeducational intervention settings.

### 2.3 Information sources and search strategy

The systematic search was conducted in the Web of Science (WoS) and Scopus databases, selected for their multidisciplinary coverage in psychology, education, child mental health, and social sciences.

The search strategy combined terms related to three conceptual nuclei:

1. Mindfulness
2. ADHD
3. Educational, psychopedagogical, or school context

The equations were applied in the title, abstract, and keyword fields, adapting the syntax to each database.

*Web of Science*: TS = ((“mindfulness” OR “mindfulness-based” OR “mindfulness intervention*” OR “mindfulness training” OR “attention awareness” OR “meditation”) AND (“ADHD” OR “attention Deficit Hyperactivity Disorder” OR “attention-deficit/hyperactivity disorder” OR “hyperactivity” OR “attention deficit”) AND (“student*” OR “child*” OR adolescen* OR youth OR school* OR classroom OR education* OR “educational setting*” OR psychopedagog* OR psychoeducational OR “special education” OR “learning support”)).

*Scopus*: TITLE-ABS-KEY ((“mindfulness” OR “mindfulness-based” OR “mindfulness intervention*” OR “mindfulness training” OR “attention awareness” OR “meditation”) AND (“ADHD” OR “attention Deficit Hyperactivity Disorder” OR “attention-deficit/hyperactivity disorder” OR “hyperactivity” OR “attention deficit”) AND (“student*” OR “child*” OR adolescen* OR youth OR school* OR classroom OR education* OR “educational setting*” OR psychopedagog* OR psychoeducational OR “special education” OR “learning support”)).

Filters were applied to include only original articles and review articles published in English or Spanish.

### 2.4 Eligibility criteria

Studies that met the following criteria were included:

#### 2.4.1 Inclusion criteria

- Original articles or reviews published in indexed scientific journals.
- Studies focused on mindfulness interventions applied to students with ADHD or related symptomatology.
- Research conducted in educational, school, psycho-pedagogical, or psychoeducational contexts.
- Studies reporting characteristics of the intervention, outcome variables, or relevant findings on attention, self-regulation, behavior, academic performance, or socio-emotional well-being.
- Documents published in English or Spanish.

#### 2.4.2 Exclusion criteria

- Editorials, letters, book chapters, theses, conference proceedings, and gray literature.
- Studies on mindfulness without relation to ADHD.
- Studies on ADHD that do not include mindfulness-based interventions.
- Research focused exclusively on adults outside the educational field.
- Documents without sufficient access to title and abstract for initial evaluation.
- Duplicate records between databases.

### 2.5 Study selection process

Records retrieved from WoS were exported in .xlsx format, while those from Scopus were downloaded in .csv format. Both datasets were integrated and processed in RStudio. First, identification and removal of duplicates were performed using the DOI as the primary identifier and, complementarily, title matching.

Subsequently, a preliminary semi-automated screening of titles and abstracts was applied using the grepl() function in R, employing key terms such as mindfulness, ADHD, attention deficit hyperactivity disorder, school, student, psychoeducational, and special education. This filtering allowed the identification of potentially relevant studies. Finally, the pre-selected documents underwent manual review of titles and abstracts to verify alignment with the inclusion criteria, thus forming the final corpus of the scoping review.

**Figure 1:**
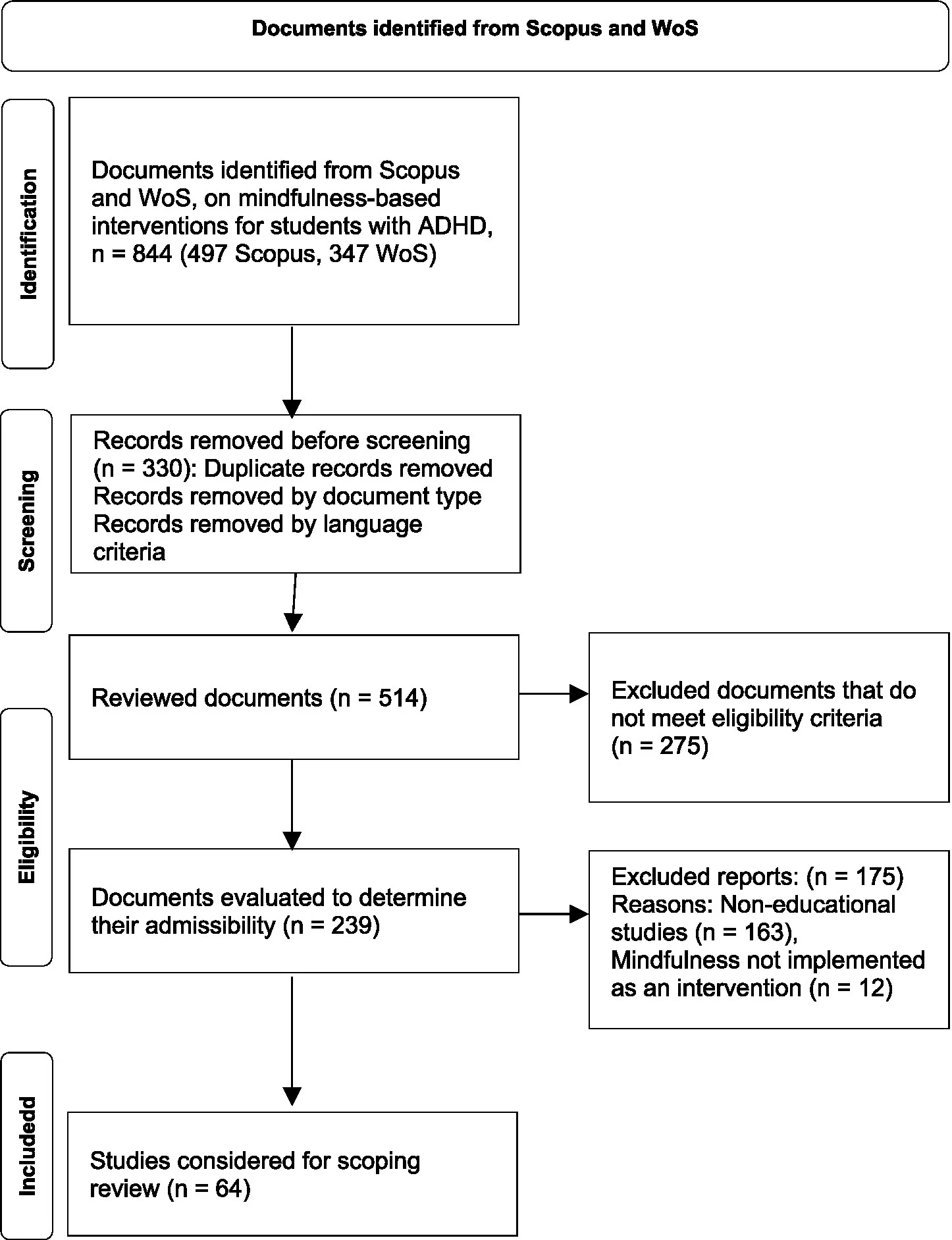
PRISMA flow diagram showing the study selection process.

The database search identified a total of 844 records, of which 497 were retrieved from Scopus and 347 from Web of Science. During the initial data-cleaning process, 330 records were removed due to duplication or because they did not meet the predefined criteria for document type and language. This resulted in 514 records being screened by title and abstract. Of these, 275 were excluded for not meeting the review criteria. The remaining 239 reports were assessed for eligibility, and 175 were subsequently excluded. Among them, 163 did not focus on educational or psycho-pedagogical contexts, while 12 did not include mindfulness as an intervention. Finally, 64 studies were included in the scoping review (Figure 1).

### 2.6 Data extraction

Data from the included studies were extracted using a structured matrix developed for this scoping review. The information collected included the study identification code, author(s) and year of publication, target population and age group, intervention or study focus, outcome measures or assessment methods, and main findings. This extraction process made it possible to compare the populations, intervention approaches, assessment strategies, and outcomes across the included studies.

### 2.7 Analysis strategy

The analysis was conducted using a descriptive, narrative, and thematic mapping approach. First, publication trends according to year, country, studied population, and methodological design type will be described. Second, the main characteristics of the mindfulness interventions will be identified, including duration, format, implementing agents, and outcome variables.

Subsequently, the studies were organized into emerging thematic categories, such as:

- Attention and executive functions.
- Emotional self-regulation.
- Behavior and hyperactivity.
- Academic performance.
- Socio-emotional skills.
- Psychological well-being.
- Application in special education or psychopedagogical support.

### 2.8 Methodological rigor considerations

Since this is a literature review based on secondary sources, ethical committee approval is not required.

## 3 Results

Table 1 presents the core scoping characteristics of the included studies, including target populations and age groups, intervention or study focus, outcome measures or assessment methods, and principal findings. This expanded mapping makes it possible to compare the populations, intervention approaches, assessment strategies, and outcomes reported across the included literature.

**Table 1:** Selected studies included in the scoping review (S1–S64).

Code	Study	Target population/age group	Intervention or study focus	Outcome measures/assessment methods	Main findings
S1	Zipkin (1985)	Students with hyperactivity and attentional difficulties	Relaxation-based techniques	Attention and hyperactivity measures	Relaxation techniques were associated with reduced hyperactivity and improved attention.
S2	Carson et al. (2001)	Students/young adults	Mindfulness-based attentional approach	Cognitive and attentional measures	Mindfulness was associated with greater cognitive flexibility and more conscious processing of stimuli.
S3	Frank et al. (2013)	School-aged students	School-based mindfulness interventions	Emotional, behavioral, and educational outcomes	School-based mindfulness interventions showed potential to strengthen emotional self-regulation and improve the learning climate.
S4	Ruiz Lázaro et al. (2014)	Children and adolescents in educational contexts	Psychoeducational and mindfulness-related strategies	Behavioral and ADHD-related outcomes	Improvements were reported in behavioral adjustment, attention-related difficulties, empathy, interpersonal relationships, and academic functioning.
S5	Pahnke et al. (2014)	Students with neurodevelopmental disorders	Acceptance- and mindfulness-related skills training	Behavioral and psychological functioning	The intervention supported behavioral and emotional regulation in students with neurodevelopmental difficulties.
S6	Murrell et al. (2015)	College students with ADHD	Mindfulness-based intervention framework	Adherence, feasibility, emotional regulation, and experiential avoidance	Mindfulness showed potential for emotional regulation, although ADHD-related characteristics created important adherence and implementation challenges.
S7	Fleming et al. (2015)	College students with ADHD	Group skills training incorporating mindfulness	ADHD symptoms, executive and behavioral outcomes	Preliminary improvements were identified in attention, self-regulation, and impulsivity-related outcomes.
S8	Waldemar et al. (2016)	Children and adolescents in public schools	Mindfulness and socio-emotional learning program	Mental health, behavior, relationships, quality of life, inattention, and hyperactivity	Improvements were found in psychosocial outcomes, but not consistently in core symptoms of inattention and hyperactivity.
S9	Singh et al. (2016)	Students with attentional difficulties	Samatha meditation	Sustained attention and academic engagement measures	The intervention increased sustained attention and improved academic engagement and performance on specific tasks.
S10	Malboeuf-Hurtubise et al. (2017)	Elementary school students with severe learning difficulties	School-based mindfulness intervention	Internalizing and externalizing symptoms, emotional regulation	The program improved emotional well-being and reduced anxiety, inattention, aggressiveness, and behavioral problems.
S11	Pérez Carmona (2017)	Adolescents with ADHD and related difficulties	Complementary and alternative approaches including mindfulness	Attention, behavior, and emotional outcomes	The evidence suggested potential benefits for attention and behavioral adjustment, although the available research remained limited.
S12	Herbert and Esparham (2017)	Children with ADHD	Mind–body interventions	ADHD symptoms and emotional regulation outcomes	Mind–body approaches showed potential to support attention and emotional self-regulation.
S13	Routhier-Martin et al. (2017)	School-aged students	Classroom-based mindfulness practices	Socio-emotional and classroom outcomes	Mindfulness practices supported socio-emotional skills and classroom adjustment.
S14	Fassbender et al. (2017b)	Adolescents with developmental disabilities	Neurocognitive assessment related to attentional control	Functional magnetic resonance imaging (fMRI)	Functional patterns in brain regions associated with attention and cognitive control were identified.
S15	Kiani et al. (2017)	Female adolescents with elevated ADHD symptoms	Mindfulness meditation training	Executive functions and emotion dysregulation measures	Mindfulness training improved executive processes and reduced emotion dysregulation.
S16	Fassbender et al. (2017a)	Adolescents with developmental disabilities	Neurocognitive assessment	Task-based fMRI and attentional performance	Neurocognitive evidence supported changes in processes related to attentional control and response monitoring.
S17	Montgomery et al. (2018)	Students from atypical populations	School-based emotional and self-regulation interventions	Emotional intelligence and school adjustment outcomes	Interventions targeting self-awareness and emotional processes showed potential for strengthening emotional functioning in educational contexts.
S18	Rosenstreich et al. (2018)	Students/young adults with attentional difficulties	Mindfulness-related assessment	Mindfulness facets, attention deficit, and postural stability measures	Greater mindfulness was associated with improved attentional awareness and reduced distraction-related difficulties.
S19	Singh et al. (2018)	Students with attentional and behavioral difficulties	Mindfulness-based practice	Self-control and academic engagement measures	The intervention improved self-control and academic engagement.
S20	Secanell and Núñez (2019)	Students with ADHD in educational settings	Educational and inclusive intervention approaches	Educational inclusion and attentional outcomes	The review identified positive educational potential but highlighted the scarcity of systematic programs designed specifically for inclusive settings.
S21	Leeth et al. (2019)	Children and adolescents with ADHD	Mindfulness interventions	Intervention objectives and self-regulation skills	Mindfulness programs primarily targeted attention, emotional regulation, self-awareness, and behavioral control.
S22	Lester and Murrell (2019)	College students with ADHD	Individualized mindfulness intervention	Stress, coping, emotional and ADHD-related outcomes	Participants showed improvements in coping and reductions in stress, although responses varied between individuals.
S23	Sibalis et al. (2019)	Young people with ADHD	Mindfulness-based intervention	Neurophysiological and attentional measures	Neurophysiological findings supported improvements in attention-related processes.
S24	Janz et al. (2019)	Young children in educational settings	Curriculum-embedded mindfulness program	Executive functioning and behavior measures	The program improved executive functioning and behavioral self-regulation.
S25	Roux and Philippot (2020)	Children and adolescents	Mindfulness-related intervention	Anxiety and behavioral outcomes	Reductions in anxiety and behavioral difficulties were reported.
S26	Santonastaso et al. (2020)	Individuals with ADHD-related psychological difficulties	Mindfulness integrated into a broader therapeutic approach	Psychological symptoms, coping, and well-being	Psychological distress decreased and coping and well-being improved.
S27	Malboeuf-Hurtubise et al. (2020)	Pre-kindergarten children	Combined philosophy and mindfulness intervention	Positive and negative mental health indicators	The combined intervention showed potential benefits for mental health and emotional functioning.
S28	Zhang et al. (2021)	Children and young people	Mental health and mindfulness-related interventions	Mental health outcomes	The evidence supported potential benefits for psychological well-being, while emphasizing variability between interventions.
S29	Bigelow et al. (2021)	Children and youth with ADHD	Acute mindfulness meditation	Executive functioning and psycho-emotional well-being	Responses differed according to cognitive profile; some participants showed improvements in executive and emotional outcomes.
S30	Kanchibhotla et al. (2021)	Adolescents	Meditation retreat	Cognitive abilities and mental and emotional well-being	Improvements were reported in cognitive functioning and emotional well-being.
S31	Malboeuf-Hurtubise et al. (2021)	Elementary school children	Online school-based intervention	Mental health and feasibility outcomes	Online delivery produced moderately positive mental health effects and demonstrated implementation feasibility.
S32	Li et al. (2022)	Teachers working with students with ADHD	Teacher-related implementation factors	Teacher attitudes and predictor measures	Contextual and professional factors influenced readiness to support students with ADHD.
S33	Guiote et al. (2022)	Children	Autogenic meditation training	Selective and sustained attention, anxiety, and mental health measures	The intervention improved selective and sustained attention, reduced anxiety, and enhanced overall mental health.
S34	Prados (2022)	Children and adolescents with ADHD	Mindfulness as an educational intervention	Attention and academic outcomes	Mindfulness was identified as a promising complementary strategy for attention and academic functioning.
S35	Porter et al. (2022)	Children and adolescents	Mindfulness-based interventions	Cognitive, emotional, behavioral, and developmental outcomes	Overall benefits were identified, but methodological heterogeneity limited definitive conclusions.
S36	Phan et al. (2022)	School-aged populations, including students with special educational needs	School-based mindfulness interventions	Evidence quality and educational/psychological outcomes	Benefits were identified across several outcomes, although evidence quality varied substantially by study design.
S37	Makmee (2022)	Elementary school students	Mindfulness training program	Attention and working memory measures	The program increased attention and working memory.
S38	Zaccari et al. (2022)	Clinical/Educational populations	Mindfulness-related intervention	General clinical and psychological functioning	Improvements in overall psychological and clinical functioning were reported.
S39	Russell and Arnold (2023)	Children and adolescents with ADHD	Complementary mindfulness-based approaches	ADHD and psychosocial outcomes	Mindfulness was supported as a complementary rather than stand-alone approach to ADHD treatment.
S40	Li et al. (2023)	Children with ADHD	Complementary therapeutic approaches	Emotional and behavioral outcomes	Complementary approaches showed potential for emotional and behavioral regulation.
S41	Virone (2023)	Young children in school settings	Early school-based intervention	School participation and self-regulation outcomes	Early intervention showed potential to improve regulation and school functioning.
S42	Kennes et al. (2023)	Adolescents	School-based mindfulness and character-strength intervention	Subjective well-being and mental health measures	The program increased subjective well-being and showed promising mental health effects.
S43	Mitsea et al. (2023)	Individuals with different mental disorders	Virtual-reality mindfulness	Cognitive, metacognitive, and psychological outcomes	Emerging technologies showed potential to extend and personalize mindfulness training.
S44	Robe and Dobrean (2023)	Children and adolescents with ADHD	Mindfulness-based cognitive training	Cardiac vagal control and core ADHD symptoms	Preliminary findings suggested short-term physiological and symptom-related effects.
S45	Ponomarev et al. (2023)	Children with ADHD	Digital cognitive training	Cognitive and language-related measures	Digital training improved selected cognitive processes, although its direct mindfulness component requires careful interpretation.
S46	Álvarez-Godos et al. (2023)	University students with ADHD	Interventions for university students with ADHD	Academic, psychological, and support-related outcomes	Existing interventions showed benefits but remained heterogeneous and insufficiently standardized.
S47	Farmer et al. (2023)	University students with ADHD traits	Mindfulness-related psychological processes	Self-compassion, emotion regulation difficulties, well-being, and distress	Self-compassion and emotion regulation were important mechanisms associated with student well-being and distress.
S48	Costa De Oliveira Martins Matos et al. (2024)	Primary school students with ADHD/teachers	Mindfulness-based socio-emotional programs	Teacher perceptions of attention, impulse control, behavior, and inclusion	Teachers perceived improvements in attention, impulse control, behavior, and educational inclusion.
S49	Jovičevič et al. (2024)	Boys aged 9–11 years with ADHD	Exercise and mindfulness program	ADHD symptom measures and physical/cognitive outcomes	The intervention reduced ADHD symptom expression in the short and longer term.
S50	Acedo Tapia and García Toro (2024)	Primary school students	Mindfulness and children’s literature	Disruptive behavior and educational outcomes	The intervention reduced disruptive behavior and supported socio-emotional functioning.
S51	Ahmed Aboalola (2024)	Young children with ADHD	Mindfulness-based intervention	Executive-function and ADHD symptom measures	The intervention improved executive functions and reduced ADHD symptoms.
S52	Seddio et al. (2024)	University students	Mindfulness-related intervention	Attention and college-health outcomes	Improvements in attention and student well-being were reported.
S53	Watroba et al. (2024)	School-aged populations	School and occupational-therapy intervention	Occupational and school-functioning outcomes	The intervention supported school participation and functional adjustment.
S54	Bazargan-Hejazi et al. (2024)	Minority pediatric populations with ADHD	Meditation modalities	Stress, ADHD-related, and health-promotion outcomes	Meditation showed potential benefits, while major evidence gaps remained for underserved populations.
S55	Tan and Jones (2024)	Adolescents	Group-based mindfulness interventions	ADHD symptoms and psychosocial outcomes	Findings were promising but mixed, and the evidence remained insufficient for firm conclusions.
S56	Kim et al. (2024)	Community youth	Mindfulness as a potential moderator of screen-time effects	Conduct problems, hyperactivity, screen time, and mindfulness	Mindfulness emerged as a potentially relevant factor in the relationship between screen exposure and behavioral difficulties.
S57	Juul et al. (2025)	Students aged 9–16 years	School-curriculum mindfulness intervention	Mental health and well-being outcomes	Large-scale school implementation provided evidence on the feasibility and longer-term effects of curriculum-based mindfulness.
S58	Dahlgren et al. (2025)	Children and young people	Mental health interventions	Mental health outcomes and evidence gaps	The review identified potential benefits but substantial gaps in follow-up and long-term evidence.
S59	Henley and Braun (2025)	Pre-service teachers	ADHD knowledge and preparation for student support	ADHD knowledge and attitudes toward supporting students	Teachers showed limited ADHD knowledge but optimism regarding their ability to support affected students.
S60	Alotaibi et al. (2026)	Twice-exceptional students	Mindfulness-based intervention	Attention-control efficiency, concentration, and executive functioning	Significant improvements were reported in attention control, concentration, and self-regulation.
S61	Seward et al. (2026)	Young people with ADHD and comorbid learning disability	Martial arts intervention with a mindfulness component	EEG/neurophysiological measures and response-inhibition tasks	The intervention helped maintain medial frontal cortex function during response inhibition.
S62	Alorani et al. (2026)	Gifted students with special learning needs	Mindfulness-based intervention	Working memory, attention, and emotional regulation measures	Improvements were reported in working memory, attention, and emotional regulation.
S63	Özbay (2026)	University students exposed to digitally intensive environments	Digital overstimulation/“brain rot” phenomenon	Qualitative interviews and thematic analysis	Digital overstimulation was associated with perceived difficulties in sustained attention, cognitive control, and well-being.
S64	Ross et al. (2026)	Children and young people/mental health populations	Mindfulness and related mental health interventions	Mental health and methodological-quality outcomes	The evidence base was growing but remained limited by heterogeneity and the need for stronger methodological rigor.

### 3.1 Publication trends and citations

**Figure 2:**
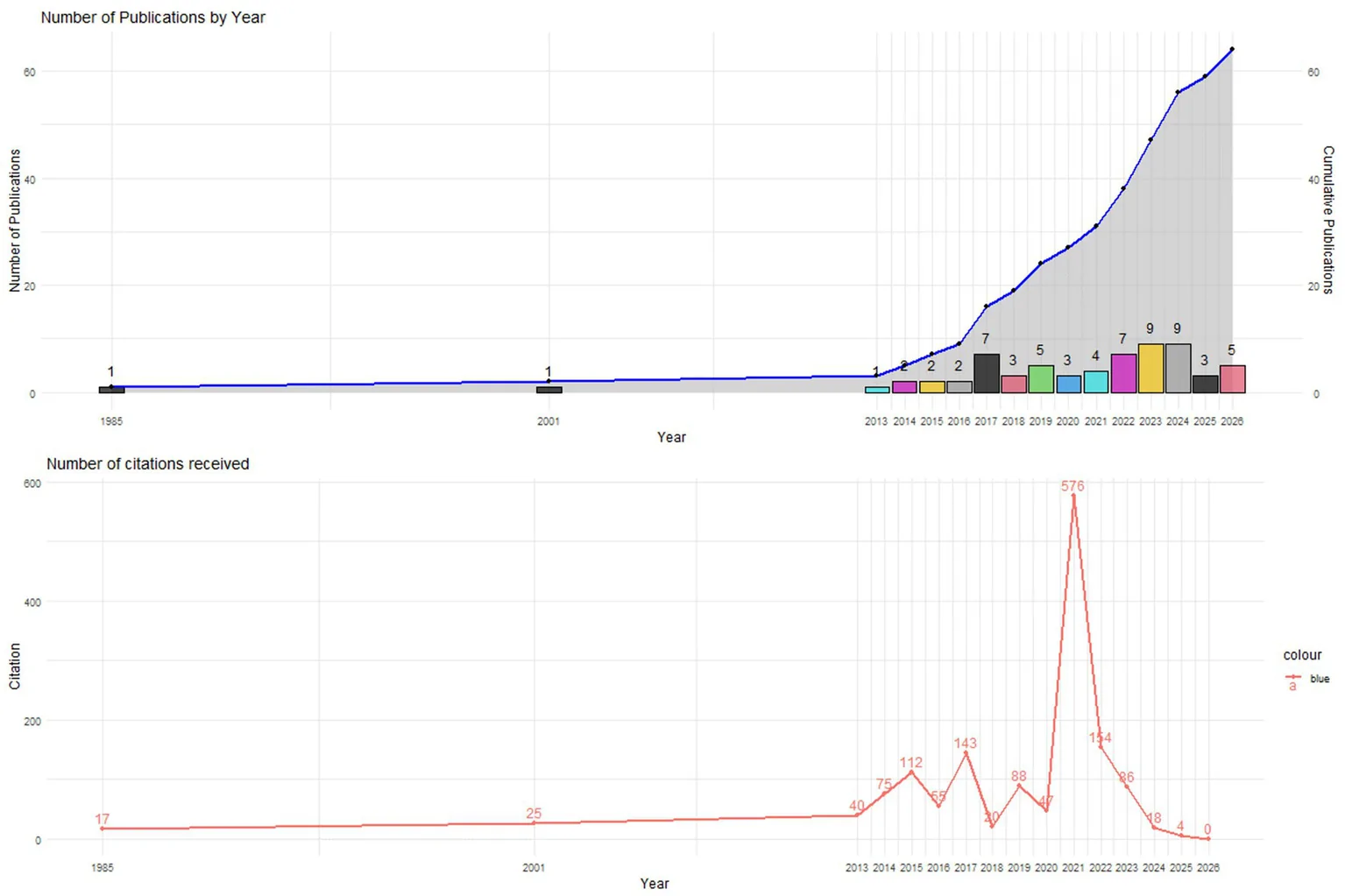
Evolution of scientific production and citation trends in mindfulness and ADHD research (1985–2026).

Figure 2 shows the temporal evolution of scientific production and academic impact in the field of mindfulness-based interventions applied to ADHD. In terms of production, incipient development is observed during the first decades (1985–2012), characterized by an almost marginal presence of publications. This pattern suggests that scientific interest in the intersection between mindfulness and ADHD was initially limited and dispersed.

From 2013 onward, an inflection point is identified, marking the beginning of a phase of sustained growth. This increase intensified progressively, reaching its peak expression between 2022 and 2024, where the highest annual production volumes were recorded. This pattern reflects the consolidation of mindfulness as a relevant approach within mental health and education research, especially in populations with neurodevelopmental disorders.

Regarding impact measured through citations, the trend shows a different behavior from production. While there was gradual growth in the early years, a pronounced peak is observed in 2022, reaching the highest number of citations recorded. This phenomenon suggests the existence of highly influential publications during that period, possibly seminal studies or reviews that have captured the attention of the scientific community.

Subsequently, a decrease in the number of citations is evident in the most recent years (2023–2026), which does not necessarily indicate a reduction in impact, but rather responds to the citation window effect. That is, the most recent articles have not yet had sufficient time to accumulate citations, a common phenomenon in bibliometric analyses.

Taken together, the figure reveals a dynamic typical of emerging fields: accelerated growth in production accompanied by a concentration of impact in certain key years. This behavior suggests that research on mindfulness and ADHD is in a phase of expansion and consolidation, with sustained growth in academic interest and progressive diversification of methodological approaches and application contexts.

### 3.2 Most influential journals

Table 2 allows the identification of differentiated patterns between scientific productivity and academic impact in the field of mindfulness-based interventions applied to ADHD. First, the journal *Mindfulness* stands out as the source with the highest number of documents (*n* = 5) and a high citation volume (141), evidencing its central role as a specialized dissemination space for this approach. This result suggests a thematic consolidation within journals specifically dedicated to mindfulness.

**Table 2:** Top 10 most influential sources by productivity and impact.

Source title	Documents	Citations
Mindfulness	5	141
Journal of attention disorders	3	126
Frontiers in psychology	3	117
NeuroImage	2	25
Journal of occupational therapy, schools, and early intervention	2	17
Journal of college student psychotherapy	2	16
Frontiers in psychiatry	2	10
British medical bulletin	1	473
Autism	1	72
Child and adolescent psychiatry and mental health	1	44

Similarly, *Journal of Attention Disorders* and *Frontiers in Psychology* present a balance between productivity (*n* = 3) and impact (126 and 117 citations, respectively), indicating that these journals not only publish relevant research in terms of quantity but also influence within the scientific community. In particular, *Journal of Attention Disorders* reinforces the direct connection with the clinical field of ADHD, while *Frontiers in Psychology* evidences the transversality of the topic in contemporary psychology.

In contrast, some journals with a lower number of publications show disproportionately high impact. This is the case of *British Medical Bulletin*, which, with a single document, reaches the highest number of citations (473), suggesting the presence of a highly influential article, possibly a reference review or study. Similarly, *Autism* (72 citations) and *Child and Adolescent Psychiatry and Mental Health* (44 citations) reflect high visibility despite low productivity, indicating that impact in this field does not depend exclusively on publication volume, but on the relevance and quality of the studies.

On the other hand, journals such as *NeuroImage* evidence the contribution of the neuroscientific approach to the field, although with lower relative impact (25 citations), suggesting that while the neurobiological component is relevant, it is not yet the dominant axis of research. Likewise, journals oriented toward educational and occupational intervention, such as *Journal of Occupational Therapy, Schools, and Early Intervention* and *Journal of College Student Psychotherapy*, present moderate levels of production and impact, reflecting a progressive integration of mindfulness in applied contexts.

Overall, the results reveal a hybrid structure of the field: on one hand, specialized journals that concentrate production, and on the other, high-impact journals that, with lower volume, generate highly influential contributions. This pattern suggests that research on mindfulness and ADHD draws from multiple disciplines—psychology, psychiatry, education, and neuroscience—consolidating itself as an expanding interdisciplinary field.

### 3.3 International collaboration networks between countries

**Figure 3:**
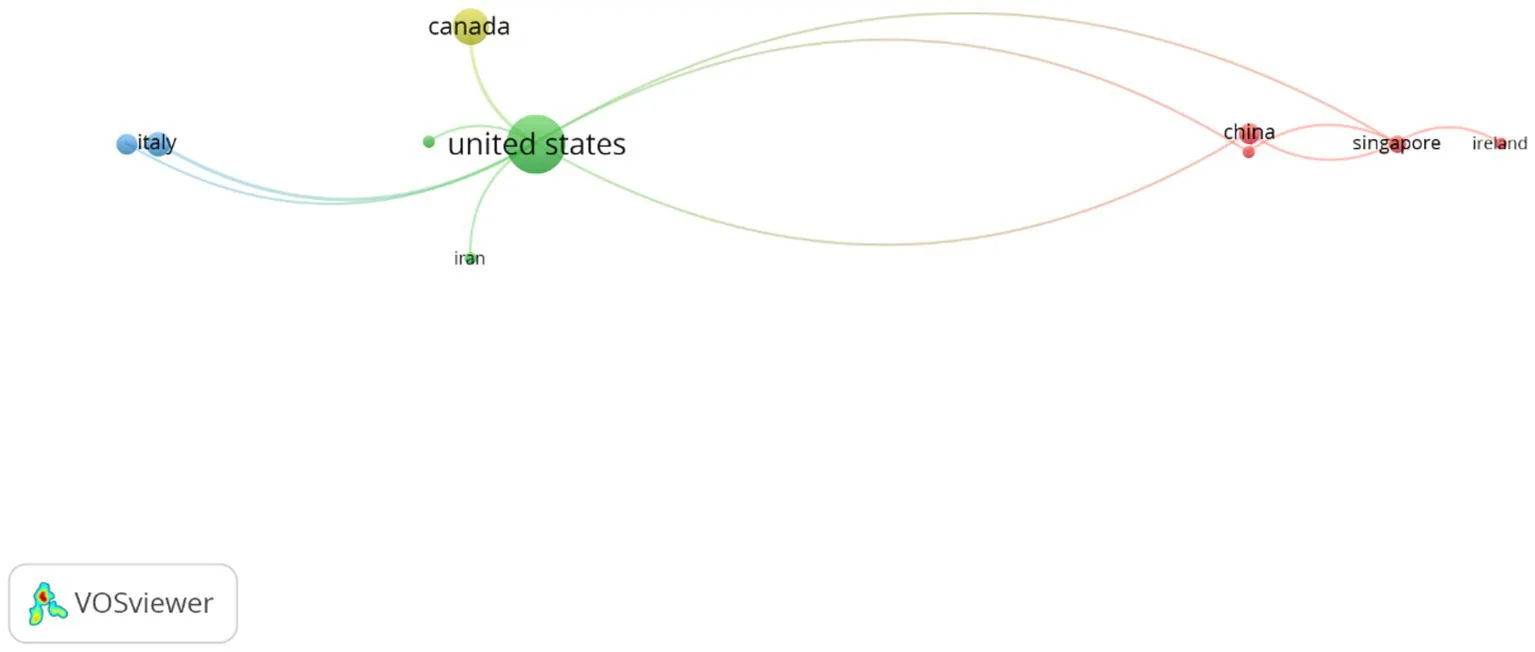
International collaboration network between countries.

Figure 3 represents the international collaboration network in scientific production on mindfulness-based interventions in the context of ADHD, evidencing a highly centralized structure with differentiated connectivity patterns between countries.

First, the United States is positioned as the central node of the network, showing the largest size and number of connections. This leadership is confirmed by the quantitative data, where it registers the highest number of documents (*n* = 22) and the highest total link strength value (TLS = 10), indicating intense international collaborative activity. This behavior suggests that the United States acts as the main articulator of knowledge in this field, connecting different geographical clusters and facilitating scientific dissemination.

At a second level, countries such as Canada, Italy, and China play relevant roles within the network, although with lower centrality. Canada stands out with 8 documents and 269 citations, evidencing not only productivity but also significant academic impact. Italy, with a link strength of 3, shows active participation in collaborative networks, while China, with 3 documents and 477 citations, is positioned as one of the countries with the highest relative impact, suggesting the presence of highly cited studies.

A relevant aspect is the formation of subnetworks or regional clusters. For example, a connection is observed between China, Singapore, and Ireland, evidencing an emerging collaboration in the Asian-European context. Singapore presents a high link strength (TLS = 4) in relation to its number of documents (*n* = 2), indicating high integration into international networks. Similarly, countries such as Italy and Iran connect with the central core led by the United States, reflecting a more dependent or peripheral collaboration.

On the other hand, several countries show marginal participation, characterized by low production and scarce connectivity (TLS = 0 or 1), such as Brazil, Belgium, Finland, or Türkiye. This dispersion suggests that, although interest in the topic is global, scientific collaboration is not yet widely consolidated in all regions.

Furthermore, it is significant that countries such as Spain (*n* = 7) present relevant production but without impact in terms of citations or strong integration into networks (TLS = 1), which could indicate limited international visibility or low collaborative articulation. Similarly, the United Kingdom and Chile show moderate levels of participation but with less structural influence in the network.

Overall, the figure reveals an asymmetrical network structure, where a reduced number of countries concentrate both production and scientific collaboration, while the rest participate in a fragmented manner. This pattern is characteristic of expanding fields, where scientific leadership is concentrated in countries with greater research infrastructure.

### 3.4 Thematic areas

**Figure 4:**
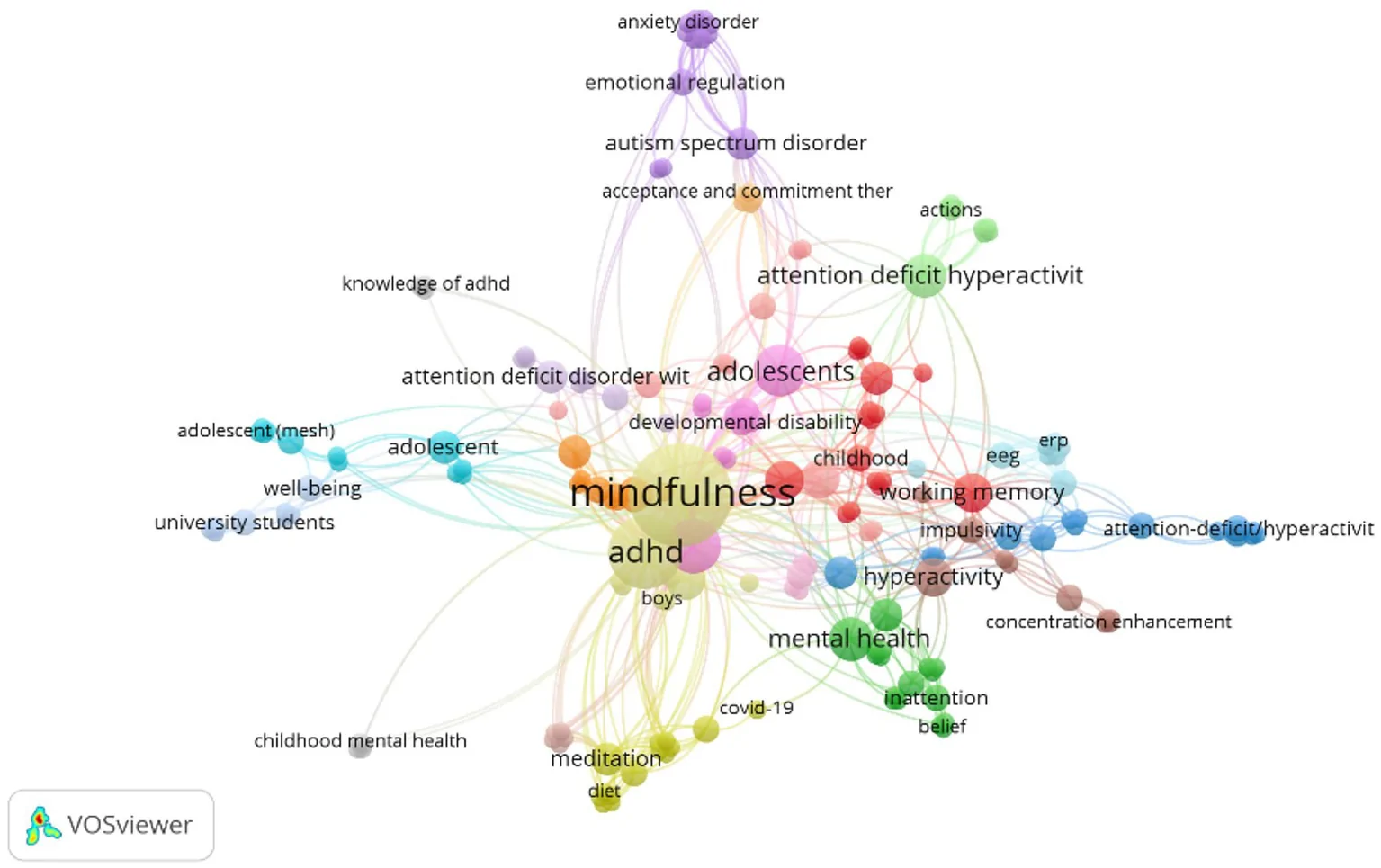
Keyword co-occurrence map of the research field.

Figure 4 represents the conceptual structure of the study field through a keyword co-occurrence map, allowing the identification of the main thematic axes, their interrelation, and the degree of consolidation of research lines around mindfulness-based interventions in ADHD.

At the core of the map, the term *mindfulness* is clearly positioned, presenting the highest frequency of occurrence (28 occurrences) and the highest total link strength value (TLS = 136), confirming its role as the articulating concept of the field. Its proximity to terms such as ADHD (14 occurrences, TLS = 66) evidences a strong interconnection between the practice of mindfulness and the study of this disorder, consolidating a well-defined research line.

A second relevant thematic axis is configured around the clinical and cognitive dimension of ADHD, where terms such as *attention*, *hyperactivity*, *impulsivity*, and *working memory* stand out. These concepts present moderate occurrence and connectivity values, indicating that research not only focuses on the intervention but also on the underlying neurocognitive mechanisms. In particular, the presence of terms such as *executive functions*, *inhibitory control*, and *EEG/fMRI* reflects a growing integration of neuroscientific approaches.

Likewise, a cluster associated with psychological well-being and mental health is identified, composed of keywords such as *mental health* (5 occurrences, TLS = 21), *emotional regulation*, and *well-being*. This group suggests that mindfulness-based interventions not only seek to improve attention but also to promote emotional balance and the integral health of students.

On the other hand, an educational and psycho-pedagogical cluster emerges, where terms such as *students*, *education*, *school-based interventions*, and *college students* appear. Although these terms present lower frequency, their connection with the central core indicates a growing application of mindfulness in formal educational contexts, especially in child and adolescent populations (*children*, 8 occurrences; *adolescents*, 7 occurrences).

Additionally, complementary therapeutic approaches are observed, such as *acceptance and commitment therapy*, *cognitive behavioral therapy*, and *yoga*, suggesting a trend toward the integration of therapeutic models within the approach to ADHD. This methodological diversification reflects the interdisciplinary nature of the field.

Finally, the appearance of emerging terms such as *digitalization*, *screen time*, and *brain rot* indicates a recent evolution of research toward the analysis of the impact of digital environments on attention, opening new lines of study at the intersection between technology, cognition, and mental health.

### 3.5 Types of mindfulness-based interventions

The analysis of the included studies evidences a clear predominance of interventions based on general mindfulness programs applied in educational and psycho-pedagogical contexts. As observed in Table 3, the majority of the research (*n* = 54) employ mindfulness training programs specifically designed for the development of mindfulness, emotional self-regulation, and behavioral control in students with ADHD.

**Table 3:** Distribution of mindfulness-based intervention types.

Type of Intervention	*n*	Studies
General mindfulness programs	54	S1, S2, S3, S5, S7, S8, S9, S10, S12, S13, S15, S16, S17, S18, S20, S21, S22, S23, S24, S25, S27, S28, S29, S30, S31, S32, S33, S34, S35, S36, S37, S38, S39, S40, S41, S42, S43, S44, S45, S46, S47, S48, S49, S50, S51, S52, S53, S54, S55, S56, S57, S58, S59, S60
Structured programs (MBSR, MBCT)	1	S61
Total analyzed	55	—

In contrast, only a reduced number of studies (*n* = 1) implement widely recognized structured protocols, such as Mindfulness-Based Stress Reduction (MBSR) or Mindfulness-Based Cognitive Therapy (MBCT). This finding suggests limited standardization in the design of interventions within the educational field, where flexible adaptation of mindfulness strategies according to the psycho-pedagogical context predominates.

Overall, the results show that the field is oriented toward applied and contextualized interventions rather than the implementation of standardized clinical models.

### 3.6 Format of intervention implementation

Regarding the format of application, the results show a significant predominance of interventions developed in group modality (*n* = 39), mainly in school or classroom settings, as presented in Table 4. This pattern reflects the psycho-pedagogical nature of the interventions, which are integrated into collective educational dynamics oriented toward learning and behavioral regulation.

**Table 4:** Formats of mindfulness intervention implementation.

Format	*n*	Studies
Group/Classroom	39	S1, S2, S3, S5, S7, S8, S10, S12, S13, S15, S16, S18, S20, S21, S22, S23, S24, S25, S27, S28, S29, S30, S31, S32, S33, S34, S35, S36, S37, S38, S39, S40, S41, S42, S43, S44, S45, S46
Individual	2	S47, S48
Total analyzed	41	—

In contrast, individual interventions are scarcely represented (*n* = 2), indicating limited exploration of personalized approaches within the analyzed literature.

These results suggest that the implementation of mindfulness in students with ADHD is mainly oriented toward group strategies, favoring its applicability in formal educational contexts.

### 3.7 Methodological designs of the studies

The analysis of methodological designs shows that, among the studies that explicitly report their approach, experimental designs predominate (*n* = 9), mainly randomized controlled trials, aimed at evaluating the effectiveness of mindfulness interventions in students with ADHD (Table 5).

**Table 5:** Methodological designs of the included studies.

Design	*N*	%	Studies
Experimental (RCT)	9	56.3%	S1, S2, S5, S7, S10, S12, S15, S18, S20
Quasi-experimental	3	18.8%	S21, S22, S23
Qualitative	4	25.0%	S24, S25, S27, S28
Total analyzed	16	100%	—

Quasi-experimental studies (*n* = 3) were also identified, representing applied approaches in real educational contexts, where experimental control is limited but an evaluative approach is maintained.

On the other hand, qualitative studies (*n* = 4) provide relevant evidence from a contextual perspective, allowing the understanding of experiences, perceptions, and processes associated with the implementation of mindfulness in psycho-pedagogical settings.

Overall, these results reflect a trend toward the use of evaluative methodologies, although with a still moderate presence of experimental designs in the field.

### 3.8 Differential pattern of outcomes: core ADHD symptoms versus broader psycho-pedagogical effects

The analysis of the included studies showed an important difference between changes in the core symptoms of ADHD and improvements in broader psycho-pedagogical outcomes. Mindfulness-based interventions did not produce the same level of effects across these two areas.

Several studies reported improvements in aspects related to educational functioning, such as emotional regulation, coping, psychological well-being, selective and sustained attention, executive functioning, inhibitory control, and academic engagement. These benefits were especially evident in studies that focused on self-regulation and psychosocial outcomes. For example, improvements in emotional regulation and executive processes were reported by Alotaibi et al. (2026), Alorani et al. (2026), Guiote et al. (2022), and Malboeuf-Hurtubise et al. (2017).

In contrast, reductions in the core symptoms of ADHD, particularly inattention and hyperactivity, were less consistent across the studies. For example, Waldemar et al. (2016) found improvements in mental health, behavior, interpersonal relationships, and quality of life, but did not report significant changes in symptoms of inattention or hyperactivity. Other studies, such as Jovičevič et al. (2024), found reductions in ADHD symptoms. These differences suggest that the effects of mindfulness may depend on factors such as the type of intervention, participant characteristics, intervention duration, and the measures used to assess outcomes.

Overall, the evidence suggests that mindfulness-based interventions may have a more consistent effect on self-regulation, emotional adjustment, executive processes, and educational participation than on the direct reduction of the core symptoms of ADHD. This distinction is important for interpreting the effectiveness of these interventions and for defining realistic psycho-pedagogical goals.

## 4 Discussion

The results of this review show that the use of mindfulness in students with ADHD has ceased to be a marginal resource and has become an intervention line with growing presence in educational contexts, although still with an uneven and methodologically heterogeneous empirical base. Rather than a consolidated field, the evidence suggests a process of transition: mindfulness no longer appears only as a complementary practice imported from the clinical field, but as a strategy that is beginning to be reconfigured from a psycho-pedagogical logic, with emphasis on self-regulation, school coexistence, and support for diverse learning trajectories.

A first relevant aspect is that the predominance of general mindfulness programs indicates that, in the educational field, these interventions have been adapted more as flexible accompaniment tools than as strictly standardized protocols. This pattern should not be interpreted solely as a methodological weakness. In reality, it appears to respond to the need to adjust practices to real school dynamics, where available time, classroom organization, and diversity of needs require malleable interventions. The project by Ruiz Lázaro et al. (2014), for example, already showed a community and school orientation in which mindfulness was articulated with health education, observing improvements in empathy, interpersonal relationships, anxiety, ADHD symptoms, and academic performance. In a similar vein, Costa De Oliveira Martins Matos et al. (2024) reported that primary school teachers perceive benefits in attention, impulse control, and behavior, in addition to considering the curricular integration of these programs desirable. In both cases, the value of mindfulness lies not only in reducing symptomatology but in its capacity to be inserted into broader educational processes.

However, the flexibility of these programs also creates challenges when comparing results across studies. The interventions are not always equivalent, which makes it difficult to determine whether the observed effects are due to mindfulness itself, its combination with other components, or the specific conditions in which the program was implemented. This issue becomes clearer when comparing studies that report improvements in specific cognitive functions with others that find broader benefits without clear changes in the core symptoms of ADHD. Alotaibi et al. (2026), for example, found significant improvements in concentration, self-regulation, and executive functioning in twice-exceptional students, while Alorani et al. (2026) reported improvements in working memory, attention, and emotional regulation. In contrast, Waldemar et al. (2016) evaluated a combined mindfulness and socio-emotional learning program in Brazilian public schools and found improvements in mental health, behavior, relationships, and quality of life, but no changes in symptoms of inattention or hyperactivity. These differences suggest that mindfulness may have more consistent effects on emotional regulation, psychosocial adjustment, and some executive processes than on the core symptoms of ADHD when these symptoms are measured more strictly.

From a psycho-pedagogical perspective, this distinction is important when defining intervention success. A student may continue to show symptoms of inattention or hyperactivity while also improving the ability to pause before reacting, recover from frustration, stay engaged in learning tasks, manage emotions, or participate more positively in classroom activities. These changes should not be considered minor outcomes, because they can affect access to learning, relationships with teachers and peers, persistence in academic tasks, and the ability to benefit from other educational supports. Therefore, mindfulness should not be evaluated only in terms of symptom reduction. In educational settings, its value may also be reflected in functional outcomes related to self-regulation, participation, coping, and adaptation to learning demands. This also supports the use of multidimensional assessment strategies that combine ADHD symptom scales with measures of executive functioning, emotional regulation, classroom engagement, academic behavior, and psychosocial adjustment.

This broader interpretation is supported by studies that reported changes in different areas of student functioning. Malboeuf-Hurtubise et al. (2017) found reductions in anxiety, depression, inattention, aggressiveness, and behavioral problems among primary school students with severe learning difficulties. Similarly, Guiote et al. (2022) reported improvements in selective and sustained attention, lower anxiety, and a better overall mental health profile. Jovičevič et al. (2024) also found lower levels of ADHD symptoms among participants who received a systematic program combining physical exercise and mindfulness, both in the short and long term. Overall, these findings suggest that the value of mindfulness in education should not be judged only by its direct clinical effects, but also by its ability to improve processes that support learning and school participation.

It is also significant that the group format is the predominant one. This reinforces the idea that mindfulness has been appropriated mainly as a classroom or group intervention, compatible with inclusive models and collective psychoeducational support strategies. The review by Tan and Jones (2024) precisely emphasizes that group interventions with adolescents present promising results, although still incipient and inconclusive. In a convergent manner, Secanell and Núñez (2019) underlined the scarcity of studies and inclusive programs specifically designed for educational contexts. This coincidence is important: the literature not only shows that the group format is the most frequent, but also that there is still a need to develop truly systematic and transferable proposals to ordinary schools. The predominance of this format appears to respond to its institutional viability, but at the same time leaves open the question of which student profiles benefit most in collective schemes and which would require more individualized or combined supports.

The methodological discussion is also central. Some recent works strengthen the empirical base by incorporating more objective measures of attentional and executive control. Seward et al. (2026), for example, showed that a martial arts intervention with a mindfulness component helped maintain medial frontal cortex function during response inhibition tasks in young people with ADHD and comorbid learning disability. This type of study is valuable because it moves the discussion beyond subjective perception and provides evidence on neurocognitive mechanisms. Nevertheless, the coexistence of pilot studies, single-group designs, narrative reviews, and works focused on teachers’ perceptions or implementation challenges indicates that the field is still building its methodological scaffolding. Murrell et al. (2015), when discussing interventions with university students with ADHD, already warned that implementation in educational contexts presents logistical, organizational, and adherence obstacles that can affect both the fidelity of the intervention and its results.

From a broader interpretive perspective, the reviewed evidence suggests that mindfulness seems to work best when integrated into programs with a clear educational rationale, and not when transferred uncritically from clinical or general well-being models. The study by Waldemar et al. (2016) is illustrative on this point, because it combines mindfulness with socio-emotional learning; that of Jovičevič et al. (2024), because it articulates it with physical exercise; and that of Costa De Oliveira Martins Matos et al. (2024), because it links it with educational inclusion. Far from diluting mindfulness, these combinations seem to show that its greatest potential emerges when it is inserted into broader psycho-pedagogical devices, oriented toward regulating behavior, strengthening emotional competencies, and sustaining school participation. This suggests that, in students with ADHD, mindfulness hardly operates as an isolated solution; its greatest value lies in being part of multimodal, ecologically valid, and context-sensitive interventions.

Consequently, the panorama offered by this review does not support a triumphalist reading nor a premature disqualification. What it shows is promising but still fragmented evidence. The available studies allow us to sustain that mindfulness has potential to favor attention, inhibitory control, emotional regulation, and some indicators of school adjustment in students with ADHD (Alorani et al., 2026; Alotaibi et al., 2026; Guiote et al., 2022; Malboeuf-Hurtubise et al., 2017; Ruiz Lázaro et al., 2014). However, the same literature also indicates that its effects are not uniform, that the reduction of core symptoms does not always appear with the same clarity, and that the consolidation of the field depends on more consistent designs, more precise descriptions of the interventions, and greater development of programs specifically designed for inclusive school environments (Secanell and Núñez, 2019; Tan and Jones, 2024). In this sense, the most solid contribution of mindfulness in psycho-pedagogy does not seem to reside in replacing other forms of support, but in expanding the repertoire of strategies to intervene on self-regulation and the educational participation of students with ADHD.

### 4.1 Mindfulness, digital overstimulation, and attention control

A recent finding from the thematic analysis concerns the relationship between ADHD, digital environments, screen time, and mindfulness. The presence of terms such as digitalization, screen time, and brain rot in the keyword co-occurrence map suggests that research is beginning to move beyond traditional clinical and school-based views of attention. There is now growing interest in the attentional demands created by highly stimulating digital environments. This issue is particularly relevant for students with ADHD, because difficulties with sustained attention, inhibitory control, and self-regulation may become more challenging in environments with constant notifications, rapid changes in stimuli, fragmented information, and frequent shifts of attention.

In this context, mindfulness may have a possible protective or compensatory role, although the available evidence is still not strong enough to confirm a direct causal effect. Mindfulness should not be understood as a direct solution to technology use. Instead, it may help students become more aware of when their attention is captured, how they engage automatically with digital content, and how difficult it may be to disengage from highly stimulating material. By training the ability to intentionally return attention to a specific task or object, mindfulness may strengthen skills that are especially useful in digitally saturated learning environments.

The study by Kim et al. (2024) is especially relevant to this emerging area because it directly connects screen time, hyperactivity, conduct problems, and mindfulness. In the same way, the appearance of digital overstimulation as a theme in this review suggests that future studies should examine whether mindfulness can influence the relationship between intensive or poorly regulated digital use and attentional or behavioral difficulties. However, this possibility should currently be viewed as an emerging research hypothesis rather than as an established therapeutic effect.

From a psycho-pedagogical perspective, this relationship also opens an important area for intervention. Mindfulness could be combined with digital self-regulation strategies, structured periods of focused work, notification management, intentional transitions between online and offline activities, and reflection on patterns of technology use. Future research should therefore go beyond measuring total screen time and examine more specific aspects, such as the type of digital activity, level of multitasking, frequency of attentional interruptions, compulsive checking behaviors, and the ability to disengage from digital stimuli. This type of research could help clarify whether mindfulness supports attentional resilience in current educational environments and whether this benefit is especially relevant for students with ADHD.

### 4.2 Limitations of the study

Despite the methodological rigor employed in this scoping review, the results should be interpreted considering certain limitations inherent to both the design and the available literature.

First, a considerable proportion of the included studies do not report in detail key characteristics of the interventions, such as duration, frequency, intensity, or implementing agents. This lack of methodological precision limits comparability between studies and hinders the identification of specific components responsible for the observed effects.

Second, significant heterogeneity is evident in the design of mindfulness interventions. The predominance of non-standardized programs, adapted to particular educational contexts, introduces variability in the results and reduces the possibility of establishing generalizable conclusions. This situation reflects both the flexibility of the approach and the absence of homogeneous implementation frameworks in the psycho-pedagogical field.

Furthermore, although studies with experimental designs are identified, the available evidence continues to be limited in terms of randomized controlled trials, which restricts the inferential solidity of the findings. The presence of pilot studies, quasi-experimental designs, and qualitative approaches, while providing contextual value, also highlights the need to strengthen methodological rigor in future research.

Another relevant limitation lies in the scarce representation of diverse geographical contexts, particularly in regions such as Latin America, which suggests a bias in scientific production toward developed countries. This concentration limits the understanding of mindfulness in educational systems with different sociocultural characteristics.

Finally, most studies focus on short-term cognitive and behavioral variables, such as attention, impulsivity, or emotional regulation, while there is limited evidence on sustained effects over time, academic performance, or educational trajectories.

### 4.3 Future research areas

The findings of this review allow the identification of multiple lines of research that require further development to consolidate the field of mindfulness in the psycho-pedagogy of ADHD.

First, it is necessary to move toward greater standardization of interventions, without losing the flexibility required in educational contexts. The development of hybrid models that integrate structured protocols with pedagogical adaptations could favor both replicability and the applicability of the programs.

Second, an increase in experimental studies with robust designs is required, particularly randomized controlled trials and longitudinal studies that allow the evaluation of the sustainability of mindfulness effects in the medium and long term.

Furthermore, it is essential to broaden the focus of analysis toward more integral educational variables, including academic performance, school retention, learning motivation, and socio-emotional development. This would allow for an understanding of the impact of mindfulness beyond the reduction of ADHD symptomatology.

Another relevant line consists of the development of personalized interventions or mixed models (group and individual), considering the heterogeneity in the manifestation of ADHD. The exploration of differentiated approaches could contribute to improving the effectiveness of interventions across different student profiles.

Additionally, the need to investigate the role of teachers as implementers of mindfulness, as well as the training and accompaniment processes required for effective implementation in the classroom, is highlighted.

Finally, the limited representation of Latin America should be addressed through a context-sensitive research agenda rather than through the simple replication of interventions developed in high-income settings. Future studies in the region should prioritize low-cost and feasible school-based interventions that can be implemented under conditions of limited psychological support services, heterogeneous school infrastructure, and substantial variation in access to digital resources. Pragmatic trials conducted in public schools would be particularly valuable for determining whether mindfulness programs remain feasible and effective under routine educational conditions rather than only in highly controlled settings.

Research should also examine how socioeconomic conditions, family demands, teacher workload, class size, rural–urban differences, and the availability of specialized support influence intervention adherence and effectiveness. Because teacher-led implementation may be more scalable than reliance on external specialists, future studies should evaluate brief training models, implementation fidelity, teacher acceptability, and the minimum level of professional support required to deliver interventions safely and consistently.

In addition, programs should undergo linguistic and cultural adaptation rather than direct translation. This includes the use of culturally appropriate examples, family and community participation when relevant, and assessment instruments with adequate validity for Spanish- and Portuguese-speaking populations. Multicenter studies involving different Latin American countries would help distinguish context-specific effects from more general intervention mechanisms. Finally, implementation research should incorporate indicators of cost, feasibility, equity, dropout, and sustainability, thereby generating evidence that is directly useful for educational systems with constrained resources.

### 4.4 Practical implications

The results of this review have relevant implications for psycho-pedagogical practice, educational management, and the design of inclusion policies.

In the educational field, mindfulness emerges as a viable tool for integration into the classroom, particularly in group format, facilitating its implementation within school dynamics without requiring significant structural modifications. Its focus on self-regulation and attention positions it as a useful resource to support students with ADHD in their learning process.

From a psycho-pedagogical perspective, mindfulness-based interventions can contribute to the development of key skills such as impulse control, emotional regulation, and concentration—fundamental aspects in the approach to ADHD. Their application in school settings also allows for preventive rather than exclusively reactive intervention.

For teachers, the evidence suggests the importance of incorporating mindfulness strategies as part of their pedagogical practice, which implies the need for training and professional development programs that enable them to implement these interventions appropriately and in context.

In the field of educational policy, the results support the inclusion of mindfulness programs within broader strategies of inclusive education and school well-being. However, their implementation must be accompanied by clear guidelines, continuous evaluation, and adaptation to the characteristics of the educational system.

## 5 Conclusion

The present scoping review evidences that mindfulness-based interventions represent an emerging and promising strategy in the psycho-pedagogical field for students with ADHD. The analyzed literature shows a growing incorporation of these practices in educational contexts, with emphasis on the improvement of attention, self-regulation, and behavior.

However, the field is characterized by high heterogeneity in the design of interventions, limited standardization, and a methodological base still under development. Although the results suggest positive effects in various dimensions, these are not entirely consistent, which highlights the need to strengthen the rigor of future research.

Mindfulness, rather than an isolated solution, is positioned as a potential component within comprehensive psycho-pedagogical approaches aimed at improving the educational experience of students with ADHD. Its greatest contribution appears to lie in its capacity to intervene in self-regulation and school adaptation processes, rather than in the direct reduction of symptomatology.

In this context, the consolidation of mindfulness as a psycho-pedagogical tool will depend on the generation of more robust evidence, the development of systematic intervention models, and its effective integration into inclusive educational practices.

## Data Availability

The original contributions presented in the study are included in the article/supplementary material, further inquiries can be directed to the corresponding author.
